# Recent applications of isotope analysis to forensic anthropology

**DOI:** 10.1080/20961790.2018.1549527

**Published:** 2019-02-17

**Authors:** Eric J. Bartelink, Lesley A. Chesson

**Affiliations:** a Department of Anthropology, California State University, Chico, CA, USA;; b Contractor at Defense POW/MIA Accounting Agency (DPAA) Laboratory, Pacific Architects and Engineers (PAE), HI, USA

**Keywords:** Forensic sciences, forensic anthropology, stable isotope analysis, isoscapes, provenancing

## Abstract

Isotope analysis has become an increasingly valuable tool in forensic anthropology casework over the past decade. Modern-day isotopic investigations on human remains have integrated the use of multi-isotope profiles (e.g. C, N, O, H, S, Sr, and Pb) as well as isotopic landscapes (“isoscapes”) from multiple body tissues (e.g. teeth, bone, hair, and nails) to predict possible region-of-origin of unidentified human remains. Together, data from various isotope analyses provide additional lines of evidence for human identification, including a decedent’s possible region-of-birth, long-term adult residence, recent travel history, and dietary choices. Here, we present the basic principles of isotope analysis and provide a brief overview of instrumentation, analytical standards, sample selection, and sample quality measures. Finally, we present case studies that reflect the diverse applications of isotope analysis to the medicolegal system before describing some future research directions. As shown herein, isotope analysis is a flexible and powerful geolocation tool that can provide new investigative leads for unidentified human remains cases.

## Introduction

Forensic anthropologists routinely examine human skeletal remains on behalf of medicolegal authorities. Most often, this involves determination of medicolegal significance, construction of biological profiles, assessment of antemortem conditions, estimation of time since death, analysis of postmortem alterations, and analysis of perimortem trauma [1]. Thus, forensic anthropologists play a key role in personal identification by providing information on sex, age-at-death, ancestry, and stature as well as identifying information on anomalies, antemortem trauma, medical intervention, and pathological conditions. While this information aids in narrowing missing persons searches, personal identification is usually achieved using more in-depth scientific evaluations, such as comparative analysis of fingerprints, DNA profiles, dentition, and radiographs. These modalities have been highly effective in identifying unknown decedents, and recent improvements in the underlying science are encouraging [2–5]. Despite the analytical power of these traditional tools, they all rely on having antemortem records with which to compare postmortem data. For longstanding unidentified remains cases, investigators increasingly turn to newer scientific tools to provide investigative leads. One area that has demonstrated promise for forensic human identification over the past decade is isotope analysis.

Isotope analysis has a long history of application in the study of ecological, geological, and hydrological systems [6]. More recently, it has also become an increasingly valuable tool in the forensic sciences, with applications ranging from analysis of explosives and other forms of trace evidence (e.g. paint, tape, and soils), sourcing and authentication of foods and beverages, and provenancing (sourcing) poached wildlife and unidentified human remains. In this article we focus specifically on the emerging role of isotope analysis for provenancing unidentified human remains to aid in resolving medicolegal cases. We first review the basic principles of isotope analysis, then briefly discuss instrumentation, analytical standards, sample selection, and sample quality measures. We conclude with case studies that reflect diverse applications of isotope analysis to the medicolegal system and also future directions. We refer the interested reader to more extensive literature reviews on forensic applications of isotope analysis [7–16].

## Isotope natural abundance

### Basic principles

Most elements in the periodic table exist in multiple forms called *isotopes*. The term “isotope” was coined in 1913 by Frederick Soddy, an English radiochemist [17]. It means “same place” and refers to the fact that all isotopes of a particular element share the same number of protons and thus appear at the same position in the periodic table.

Isotopes of an element are distinguished by differences in the number of neutrons. Of all the elements in the periodic table, only 21 are monoisotopic, meaning they have a single naturally occurring isotope form [17]. The stable isotope forms of an element typically include one common isotope and one or more rare isotope(s). For instance, ∼98.89% of the element carbon is the stable isotope ^12^C while ∼1.11% is the stable isotope ^13^C. The superscript notation used for identifying isotopes represents the sum of protons and neutrons – e.g. ^12^C has 6 protons and 6 neutrons, while ^13^C has an additional neutron and is slightly heavier. Unlike radioactive isotopes (e.g. ^14^C with two extra neutrons), stable isotopes do not decay over time.

Isotopic compositions of non-metal elements (such as H, C, N, O, and S) are typically expressed as ratios (*R*) of a rare to common stable isotope (e.g. *R* = ^13^C/^12^C, ^15^N/^14^N). Since *R* is small for most stable isotope pairs, and measured differences in isotope ratios at natural abundance are also small, delta (*δ*) notation is used to express deviation of the sample *R* from a reference standard in parts per thousand (‰): *δ* = (*R*_sample_ – *R*_std_)/*R*_std_. For hydrogen (^2^H/^1^H), the isotopic composition of a sample in *δ*-notation would be expressed as the *δ*^2^H value, while for O (^18^O/^16^O) the isotopic composition of a sample would be expressed as the *δ*^18^O value. Reference standards for the non-metal elements include Vienna-Standard Mean Ocean Water (VSMOW) for H and O; Vienna-Pee Dee Belemnite (VPDB) for C – and, in some cases, O as well; Atmospheric Nitrogen (AIR) for N; and Vienna-Canyon Diablo Troilite (VCDT) for S.

Stable isotopes of interest for the metal elements strontium (Sr) and lead (Pb) include primordial stable isotopes and radiogenic stable isotopes, or isotopes that are the (stable) products of radioactive decay. The radiogenic stable isotope ^87^Sr is the product of the decay of ^87^Rb, while the radiogenic stable isotopes ^206^Pb, ^207^Pb, and ^208^Pb are products of the decay of ^238^U, ^235^U, and ^232^Th, respectively. When measured alongside primordial stable isotopes (i.e. ^86^Sr and ^204^Pb), isotopic compositions of metal elements are typically reported as *R*_sample_, with no conversion to *δ*-notation – e.g. ^87^Sr/^86^Sr, ^206^Pb/^204^Pb, ^207^Pb/^204^Pb, and ^208^Pb/^204^Pb. Although reference standards are not needed to report Sr and Pb isotope ratios in *δ-*notation, there are standard reference materials available for calibration and verification of measured Sr and Pb isotope ratios: SRM-987, a strontium carbonate reference material, and SRM-981, a common lead reference material.

### Isotopic fractionation

Both the lighter and heavier stable isotopes of an element may take part in reactions, but the small mass differences between the isotope forms cause them to behave differently in chemical and physical processes. The partitioning of elements and their isotopes between two or more substances or fractions (pools) is called *isotopic fractionation*. For two isotopes of the same element, the strength of bonds involving the heavier isotope is usually slightly greater – and thus slightly more difficult to break – than the strength of bonds involving the lighter of the two isotopes. Thus, mass-dependent isotopic fractionation can take place during chemical reactions when bonds are broken and reformed. Mass-dependent isotopic fractionation can also take place during physical processes that involve separating molecules with different masses – for example, gas exchange through plant stomata.

Consider the movement of H and O isotopes through the global water cycle as another example of mass-dependent isotopic fractionation. Water molecules of differing isotopic composition and thus differing mass – called isotopologues, and containing various combinations of the stable isotopes ^1^H, ^2^H, ^16^O, ^17^O, and ^18^O – are impacted differently by the processes of evaporation and condensation [18]. The heavier isotopologues of water tend to remain in the liquid form as compared to their lighter counterparts. As a consequence, the hydrogen and oxygen isotopic compositions of water vary systematically in space when measured as precipitation [19] or tap water [20]. There is a strong correlation between the isotopic composition of water and atmospheric temperature, elevation, and distance from the coast. Water molecules containing ^1^H or ^16^O are generally found further inland in cooler climates, and further along gradients of temperature, altitude, and latitude, than water molecules containing ^2^H or ^18^O.

## Forensic applications of isotope analysis

Applications of isotope analysis have a long history in chemistry, biology, ecology, geology, hydrology, oceanography, archaeology, and physical anthropology [15]. However, in comparison, the history of applications of isotope analysis for provenancing unidentified human remains from medicolegal cases is relatively short. Over the past decade, predictive models have been developed to provide parameters from which to compare isotope values in animal tissues, including bird feathers, teeth, bone, hair, and nails. Most commonly, predictive models incorporate spatial information (i.e. via geographic information system (GIS)) to generate reference isotopic landscape maps, or “isoscapes” [15,21,22]. Isoscapes can be generated for multiple materials (e.g. water and soil) and tissues (e.g. teeth, bone, hair, and nails) for a number of isotope systems (e.g. C, N, O, S, Sr, and Pb). Predictive isoscape maps are especially useful for multi-isotope applications where overlapping isotopic profiles can aid in narrowing regions-of-origin. In conjunction with the biological profile and other identifying information from the skeleton, isotope analyses can provide new investigative leads by narrowing down possible geographic regions from which a person traveled or previously lived.

## Isotopes used for human provenancing

### Bio-elements

Since the recognition of the basic principles of isotope geochemistry in 1925, research and analysis has focused primarily on five non-metal elements and their isotopes: H, C, N, O, and S [17]. These five, the so-called “bio-elements”, make up most of the tissues in living organisms.

#### Hydrogen and oxygen

The H and O isotopic compositions of human tissues – such as bone or hair – can be related to the *δ*^2^H and *δ*^18^O values of water consumed by an individual, either directly as drinking water and other beverages or indirectly via food. Because the *δ*^2^H and *δ*^18^O values of water vary geographically, it is possible to measure the isotopic composition of a tissue, predict the related drinking water using an empirically determined equation, and thus generate a prediction on the region-of-origin of an individual in the period of life represented by that tissue – for example, hair [21]. While these origin predictions based on estimated drinking water isotopic composition often cover large areas, or bands, of regions with similar climate, they can be very useful in excluding source areas from consideration to “reduce the haystack” of possible origins of an individual [7].

Determining the “best” calibration data to use when relating the isotopic composition of a tissue to drinking water is not always straightforward and model validation is an area of active research. For example, there are multiple regression equations published to convert bone bioapatite *δ*^18^O values to drinking water *δ*^18^O values – i.e. [23–27]. Recent work by Pollard et al. [28] suggests that it may be better to predict origin based on a direct comparison of measured samples to exhibits of known origins, although to date there have been very few isotope datasets of human tissues published for this type of sample-to-sample comparison. Significant oxygen isotope datasets of particular note include a large collection of dental remains from U.S. Americans and Asians published by Regan [29] and smaller collections of dental remains from Japan published by Someda et al. [30] and the Middle East published by Posey [31]. Raynaud [32] published a large dataset of *δ*^2^H and *δ*^18^O values of human hair.

However, extreme caution is needed when using published isotope data of human tissues to create composite datasets for calibration models or to make direct sample-to-sample comparisons for provenancing. Sample preparation method and/or analysis technique can significantly impact measured *δ*-values. This was recently and vividly demonstrated by Pestle et al. [33] using multiple sub-samples of the same archaeological femur, which revealed “significant inter-laboratory isotopic variation for both collagen and carbonate (abstract, p.1)” most notably in the *δ*^18^O values of bioapatite. Those authors found that approximately half of the isotopic variability observed between laboratories was due to differences in sample preparation procedure while the other half was due to differences in isotope analysis technique. As an additional warning note, the hydrogen isotope analysis of proteinaceous tissues, such as hair, is difficult due to the presence of labile H atoms, which must be controlled and accounted for during sample preparation and analysis [34,35].

#### Carbon, nitrogen, and sulfur

Sometimes referred to as the “dietary” elements, the isotope analyses of C, N, and S provide information on the foods consumed by an individual. When food consumption patterns vary due to cultural preference or features of local geography, the *δ*^13^C, *δ*^15^N, and *δ*^34^S values of human tissues may also provide some geolocational information about different populations. As noted previously for H and O, the isotope analysis of C, N, and/or S can be useful for excluding potential origins of an individual from consideration. For example, the *δ*^13^C and *δ*^15^N values of bone collagen can be useful for discriminating the remains of U.S. American service members from local Asians [36] as well as U.S. Americans from undocumented border crossers from Latin America.

Carbon isotopic variation in human tissues is empirically related to an individual’s diet of plants and animals that obtain their carbon primarily through one of two major photosynthetic pathways: C_3_ and C_4_. Plants using C_3_ photosynthesis – which include rice, wheat, fruit trees, and most other cultivated crops – are characterized by *δ*^13^C values ranging from –35‰ to –20‰ [37]. In contrast, C_4_ plants are characterized by *δ*^13^C values ranging from –14‰ to –10‰ and include two crops of particular note for modern human nutrition, maize (corn) and sugar cane. The tissues of individuals consuming significant quantities of C_4_ plants, either directly through plant-based foods or indirectly through meat raised on C_4_ fodder, will be isotopically distinct from those of individuals consuming primarily C_3_-based foods. (We note that significant intake of marine-based foods will also have an impact on the carbon isotopic composition of a tissue, as marine resources are typically elevated in ^13^C relative to terrestrial resources). It is perhaps not surprising that archaeologists have been using carbon isotope analysis for decades to reconstruct palaeodiets [38–42]. Even today, when similar foods are available globally and diets are becoming more homogenized (the so-called “supermarket diet” [43]), there exists geographic patterning in *δ*^13^C values of human hair across the globe [44,45].

Variation in *δ*^15^N values of human tissues is related to protein consumption, with the tissues of individuals that consume higher quantity (or higher quality [46]) of protein typically characterized by higher *δ*^15^N values. This is often referred to as the “trophic level effect” [47] and can be summarized more generally as, “You are what you eat… plus a few permil” [38]. Most studies show *δ*^15^N values elevated by 2‰–4‰ in consumer’s tissues over their main source of dietary protein [48,49]. Some metabolic conditions also affect the *δ*^15^N values of tissues. For instance, patients suffering from eating disorders may have higher-than-expected *δ*^15^N values of hair due to the breakdown and reprocessing of body proteins during starvation conditions [50,51]. It is thus advisable to carefully interpret the nitrogen isotopic compositions of human tissues in conjunction with the carbon isotopic compositions.

To date, analysis of ^34^S/^32^S ratios in human tissues for human provenancing has been underutilized as compared to analysis of ^13^C/^12^C and ^15^N/^14^N ratios. This may have to do with the lower abundance of sulfur as opposed to carbon and nitrogen in organic materials, requiring larger sample sizes for analysis. However, recent advances in elemental analysis with isotope ratio mass spectrometry (IRMS) have made it possible to simultaneously measure the *δ*^13^C, *δ*^15^N, and *δ*^34^S values of organic materials [52–55]. An additional roadblock to more widespread use of sulfur isotope analysis may be data interpretation, with the factors affecting sulfur isotopic compositions of human tissues generally not as well understood as those impacting carbon and nitrogen [56], plus the role of pollution affecting *δ*^34^S values of modern ecosystems [57]. The *δ*^34^S values of human tissues, such as bone collagen or hair keratin, are most significantly impacted by marine influences – either through direct consumption of marine-sourced foods or cultivation of land located near coastlines and impacted by marine aerosols (the “sea spray effect”). Because marine *δ*^34^S values are higher than those observed in terrestrial settings [58], higher *δ*^34^S values of a human tissue are typically interpreted as indicative of an individual’s increased reliance on or proximity to marine resources.

### Trace metal elements

In addition to the five bio-elements described previously, two metal elements found in lower (trace) concentrations within human tissues have been used for human provenancing: strontium (Sr) and lead (Pb). Application of these trace metals in forensic anthropology is not nearly as widespread as the bio-elements, most likely because there are fewer laboratories offering isotope analysis of Sr and/or Pb. The extremely small differences in the masses of trace element isotopes require more precise isotope ratio measurements – and thus more expensive instrumentation – than what is needed for measurements of the isotope ratios of the bio-elements. Sample preparation requires a dedicated clean room (or rooms) while the instrumentation used for trace element isotope analysis is not only expensive but requires significant training to operate. With that said, isotope analysis of Sr and Pb is often helpful in forensic provenancing as it can “reduce the haystack” of potential sources even more than analysis of the bio-elements alone.

#### Strontium

Sr isotopic variation in the environment is ultimately related to a bedrock deposit, its age, and its susceptibility to weathering [59]. As noted previously, the radiogenic isotope ^87^Sr is a product of the radioactive decay of ^87^Rb. Bedrock deposits with high initial concentrations of ^87^Rb and/or deposits that are old typically have higher ^87^Sr/^86^Sr ratios than young deposits or deposits with low concentrations of ^87^Rb. Weathering releases Sr from bedrock into the local environment where it is taken up by plants and then ingested by animals in their food.

Recently, maps of predicted Sr isotopic variation in the environment have been published for the U.S. [22,60] as well as selected world regions [61–66]. Within humans, Sr can substitute for calcium and is found primarily in bones and teeth. There is in essence no isotopic fractionation between Sr in source (e.g. food and local environment) and target (e.g. tissue) [67]. Thus, Sr isotope analysis of human tissues can be used to reconstruct the potential travel movement history of individuals [22,68,69] through comparison of measured ^87^Sr/^86^Sr ratios in a sample to published Sr isoscapes or other reference sample datasets.

#### Lead

Similar to Sr, the lead isotope method [70] takes advantage of radioactive decay, time, and the relative concentrations of elements (specifically Pb, Th, and U) in the environment. The radiogenic isotopes ^206^Pb, ^207^Pb, and ^208^Pb are derived from the radioactive decay of ^238^U, ^235^U, and ^232^Th, respectively. Variations in the ratios of ^208^Pb/^204^Pb, ^207^Pb/^204^Pb, and ^206^Pb/^204^Pb are thus related to the geologic age of Pb ores [70]. Anthropogenic activities can also impact Pb isotopic variation in the environment, such as ore mining or the introduction of tetraethyllead in gasoline in the 1920s as an “antiknock” agent for engines. These anthropogenic factors must be considered when creating reference samples datasets or Pb isoscapes [71] to ensure both spatial and temporal comparisons are appropriate for the provenancing questions being asked.

At present there are relatively few published Pb isoscapes, for the U.S. and Europe [71,72]. However, additional reference datasets of Pb isotopic variations in human tissues are available, e.g. [29,69,73,74]. Like Sr, Pb can substitute for calcium in bone and teeth and there is generally no isotopic fractionation between element source and human tissue.

## Instrumentation and standards

### Brief overview of instrumentation

Although anthropological and archaeological applications of isotope analysis have expanded greatly since the first pioneering studies in the 1970s on palaeodiet, and more investigators than ever rely on the technique to understand human behaviors, for some users of isotope data the act of analysis itself is a bit of a “black box”: samples go in and results come out. In the space available for this article it is impossible for us to provide a detailed explanation of isotope analysis and the available methods, instrumentation, etc. Instead, we provide a brief overview of some common types of instrumentation and highlight the importance of standards in analysis. For more detail the reader is highly encouraged to review the Forensic Isotope Ratio Mass Spectrometry Network’s *Good Practice Guide for Isotope Ratio Mass Spectrometry* [75], which is freely available online (http://www.forensic-isotopes.org), as well as other resources [7,76,77].

#### Isotope analysis of bio-elements

The isotope ratios of the bio-elements are typically measured via IRMS. Samples must be converted into simple gases for analysis: i.e. H as H_2_, C as CO_2_, N as N_2_, O as CO, and S as SO_2_. Historically, samples were converted “off-line” and the purified sample gases were introduced to the mass spectrometer alternatively with a reference gas of known isotopic composition in what is known as dual inlet-IRMS. Today, most samples are converted to gas immediately before introduction to the mass spectrometer through the use of various peripherals and a carrier gas (He) in what is known as continuous flow-IRMS (CF-IRMS). In CF-IRMS, the working gas is analysed once, either just before or just after the sample gas.

**Figure 1.**
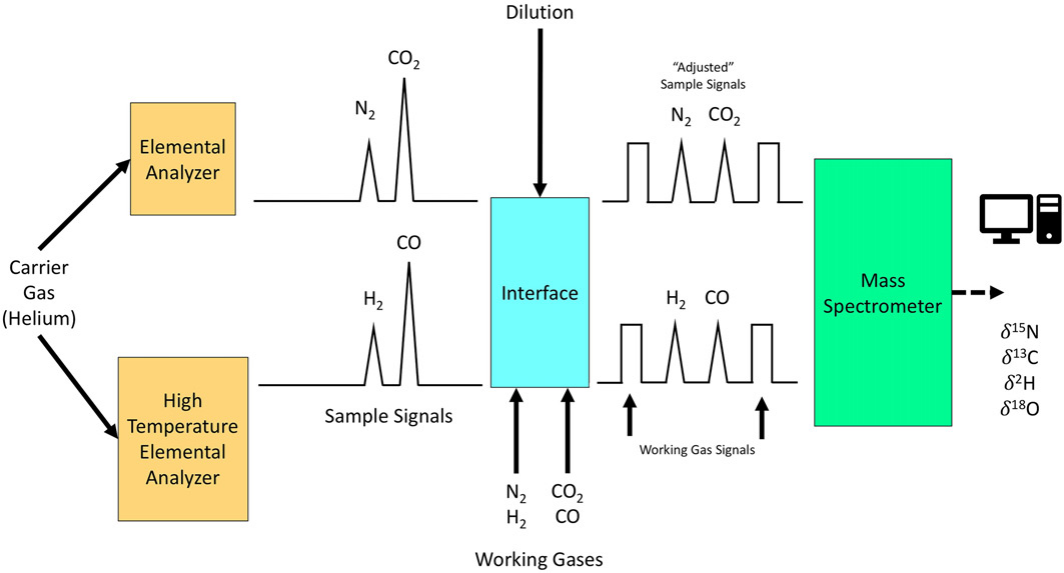
A simple schematic of the analysis of bio-elements (e.g. H, O, C, N) via continuous flow-isotope ratio mass spectrometry (CF-IRMS). Redrawn from [78] with permission granted by the FIRMS’ Board of Directors.

Sample analysis via CF-IRMS can be divided into four steps: (1) Combustion or thermal conversion of a sample into simple gases using an elemental analyzer (EA); (2) introduction of the gases into the ion source of the mass spectrometer via the interface; (3) ionization of the gas molecules followed by separation and detection of the ions in the mass spectrometer; and (4) evaluation of the raw data. The gases N_2_, CO_2_, and SO_2_ are produced by combustion using an EA while H_2_ and CO gases are produced using a high temperature conversion (HTC), or pyrolysis plus EA. A simple schematic diagram of a CF-IRMS system is presented in Figure 1 [78]. To ensure all isotopes of an element are counted, multiple masses must be monitored simultaneously – for example, N_2_ containing ^14^N^14^N (mass 28) versus ^14^N^15^N (mass 29). The working gas measured just before or after the sample is used to convert *R*_sample_ into a preliminary *δ*-value, sometimes called a “raw” *δ*-value.

Recently a new method for isotope analysis of bio-elements has been introduced: isotope ratio infrared spectroscopy (IRIS) [79–81]. The technique relies on the characteristic absorption of light by different isotopologues of gas to measure isotope ratios. At present, IRIS analysis is limited to H, C, N, and O isotope analysis of primarily atmospheric gases (e.g. carbon dioxide, methane, water, etc.).

#### Isotope analysis of trace metal elements

The isotope ratios of the trace elements can be measured via thermal ionization mass spectrometry (TIMS) or multi-collector inductively coupled plasma mass spectrometry (MC-ICP-MS). While TIMS is the classic trace element isotope analysis technique, MC-ICP-MS overtook TIMS in popularity 2 decades ago as it is possible to analyse more elements’ isotope ratios at lower cost and faster with equivalent or better precision [70,82]. Isotope analysis via MC-ICP-MS involves the ionization of a sample (usually in liquid form) through the use of a high-energy plasma discharge. Ions of different isotopes – for example ^86^Sr^+^ and ^87^Sr^+^ – generate different mass spectra that are measured by an array of ion collectors [83]. For simple schematic diagrams and comparisons of several commercially-available MC-ICP-MS instruments, the reader is referred to Figure 3 in Ref. [77], P.1176.

As noted previously, the abundances of different isotopes of Sr and Pb are typically reported as ratios (*R*_sample_), with no conversion to *δ*-notation as used in the reporting of bio-element isotopic compositions. As such, no standard is required to report trace element isotope ratios on an isotope ratio *δ* scale. However, most analysts include an international reference material, such as SRM-987, in analytical sequences for quality control purposes – i.e. to demonstrate that the measured *R* for the standard is within uncertainty of its assigned *R*.

#### Standard calibration and quality assurance

To place the “raw” *δ*-values measured via IRMS in context – and to allow comparison of data with other laboratories – reference materials are used to calibrate measurement results and report them on the appropriate isotope ratio *δ* scale. For this reason, reference materials/standards *must* be measured alongside samples in every analysis. The *δ* scales are defined by primary (calibration) materials, many of which no longer exist. Secondary (reference) materials have been carefully calibrated to the primary materials; these secondary materials are available for purchase by laboratories, but typically in limited quantities to prevent their exhaustion. For day-to-day operation, analysts will typically develop in-house (laboratory) standards that are calibrated to the secondary reference materials.

IRMS data handling procedures for measured *δ*-values will vary from laboratory to laboratory, but there are some commonalities. The “raw” *δ*-values of replicates of the laboratory standards included in an analytical sequence can be reviewed for variability based on position in the sequence (drift or time correction) and size (area or mass correction). In some cases, the isotopic composition of the sample analysed prior to a standard will affect the measured *δ*-value and a memory correction is needed. For nitrogen isotope analysis in particular, a blank correction may be needed to account for the influence of trace amounts of N in the empty sample capsules themselves and/or atmospheric N_2_. Finally, data are normalized to convert “raw” *δ*-values of samples to the “true” *δ*-values reported on the appropriate isotope ratio *δ* scale. Historically normalization was calculated as a one-point or offset correction based on multiple measurements of a single laboratory standard. Today, recommended good practice for forensic isotope analysis advocates the use of at least two laboratory standards for normalization to generate a stretch-shift or slope-intercept correction [75,84,85].

It should be obvious by now that many aspects of isotope sample preparation and IRMS analysis can potentially affect the *δ*-values reported. To address this, some recent publications have presented recommended terminology and data presentation guidelines for reporting the results of bio-element isotope analysis. These include recommendations for forensics [84], ecology [86], and archaeology [87,88]. To date, however, we are unaware of any published guidelines for reporting the results of trace metal isotope analysis.

## Sample selection and quality measures

While isotope ratios can be measured in all human tissues and bodily fluids, forensic applications are often constrained by the tissues available for sampling as well as the specific needs of a particular case investigation. Time and funding are additional factors that may constrain the types and extent of isotope analysis completed.

Different tissue types further provide different snapshots of time, such as childhood diet and residence (teeth), diet and location for the past 10–20 years of life (bone), and more recent travel history or residence patterns (hair and nails). From an investigative standpoint, hair and/or nails may be the most useful because they can provide information on life history just prior to death. However, in unidentified remains cases, hair and nails may not preserve or be retained following autopsy or skeletal analysis, leaving only teeth and bone available for sampling. Hair and nail keratin and bone collagen are the most commonly sampled proteinaceous tissues, whereas bioapatite from teeth and bone is the most commonly sampled biomineral tissue.

### Hair and nail keratin

Hair and nails are considered serial recorders in that they provide an incremental record of recent diet and residence. For many modern-day unidentified remains cases, keratin from hair and nails is often preserved and can be used to reconstruct a decedent’s residence patterns and dietary choices. Although various factors such as health and age can influence growth rates of keratin, hair grows on average 0.4 mm per day [89] and fingernails and toenails about 2–3 mm and 1 mm per month, respectively [90]. Sub-samples of hair and nails can be used to reconstruct a travel and dietary history for an unidentified decedent. Individuals who are sedentary may show similar isotope ratios throughout serial sections of hair or nails, whereas a traveler may show values that change regularly. For incremental studies of hair and nails, it is important to record the directionality of the tissue so that changes in isotopic composition can be examined chronologically. Hair is the preferred sample over nails given that its growth rate and metabolic inputs are better understood.

### Bone collagen

Bone tissue is constantly remodeled throughout life. However, the remodeling rate is not constant and varies throughout the lifespan, with faster bone turnover times in young adults compared to elderly adults. Bones within the skeleton also remodel at different rates. For example, a rib may provide an archive of residence and diet representing the last 5–10 years of life whereas a femur may provide a record of the last 20–25 years of life [7,91]. Bone is composed of both collagenous and non-collagenous proteins (about 35% by weight) and biominerals (about 60% by weight) [92]. Collagen is composed of both nonessential and essential amino acids, the latter of which derive from dietary protein [93,94].

### Bioapatite: teeth and bones

Hydroxyapatite, or bioapatite, is the biomineral component of tooth enamel and bone. While both the carbonate and phosphate fractions can be analyzed, the former is preferred due to its straightforward sample preparation procedures. Unlike carbon isotopes of bone collagen, which are biased toward dietary protein, bioapatite forms from dissolved bicarbonate in the blood and provides a record of consumption of carbohydrates, lipids, and protein not used in tissue synthesis of bone collagen (i.e. bioapatite reflects whole diet) [93,94]. In addition, oxygen isotopes of the carbonate in bioapatite reflect residence patterns during the time of tissue formation. Thus, oxygen isotopes of teeth reflect region-of-origin during infancy and childhood, whereas bone provides a record of residence history during the last several years of life. Bioapatite in bone can potentially produce misleading results due to turnover and mixed residence pattern signals. However, the data will be useful for cases where an individual died recently after migrating or traveling to a new area since their isotope profiles will still reflect their previous residence location.

### Diagenesis

In context of unidentified human remains cases, diagenesis can be defined as the chemical alteration of biological remains due to the interaction with the environment, including soil and groundwater [9]. For most cases, diagenesis is not a major concern as the hard tissues of the skeleton are resilient against chemical alteration for short time intervals (usually days to months after death). However, for human remains that have been exposed or buried for years to decades, significant degradation may occur, especially to hair and nails, and to a lesser degree, bone and teeth. Sample quality assessment metrics established for keratin, collagen, and bioapatite provide parameters for including or excluding samples for isotope analysis [8]. Evaluation of sample quality is critical for medicolegal cases since compromised samples may yield inaccurate and misleading results.

## Case studies

### Provenancing human remains

In this section, we present the application of isotope analysis as an investigative tool using three case examples. We emphasise the use of different isotope systems for answering specific questions and for predicting region-of-origin of unidentified human remains cases and deceased undocumented border crossers from the U.S.-Mexico border.

#### Case Study 1: human versus nonhuman determination

In 2016, a long bone shaft fragment was recovered during subsurface testing at a construction site in downtown San Francisco. The bone was examined by a local cultural resource management firm, and an archaeologist identified it as possibly human. The construction company and medical examiner were concerned that the bone may be Native American in origin, causing potential issues with future development at the site. The bone fragment was sent to the Human Identification Laboratory (HIL) at California State University, Chico for macroscopic analysis. The bone had been fragmented into eight pieces and had to be reconstructed. Based on overall morphology, the fragments derived from a mammalian long bone, such as a humerus, tibia, or femur. The overall texture and distribution of cortical and cancellous bone compared more favorably with human bone than nonhuman bone. For example, the boundary between cortical and cancellous bone is poorly defined in humans whereas in nonhuman animals, it is a much more defined boundary [95]. Although the macroscopic assessment did not permit a definitive identification as human, it was more consistent with being human than nonhuman.

For a more conclusive determination, the sample was submitted to Forensic Anthropology Center at Texas State (FACTS) for histological examination. In humans, the bone microstructure typically appears as a series of concentric secondary osteons. In most nonhuman animals, the bone microstructure appears as a series of brick-like structures, characteristic of plexiform bone [96,97]. A small sample of bone was embedded in resin and slides were prepared using standard histological protocols and examined using an Olympus CX41 microscope (Olympus, Tokyo, Japan). During the histological examination, both lamellar bone and osteonal bone were noted as well as a lack of osteonal banding, negating a definitive determination of human versus nonhuman. The degraded nature of the bone was a limiting role in the histological assessment.

**Figure 2.**
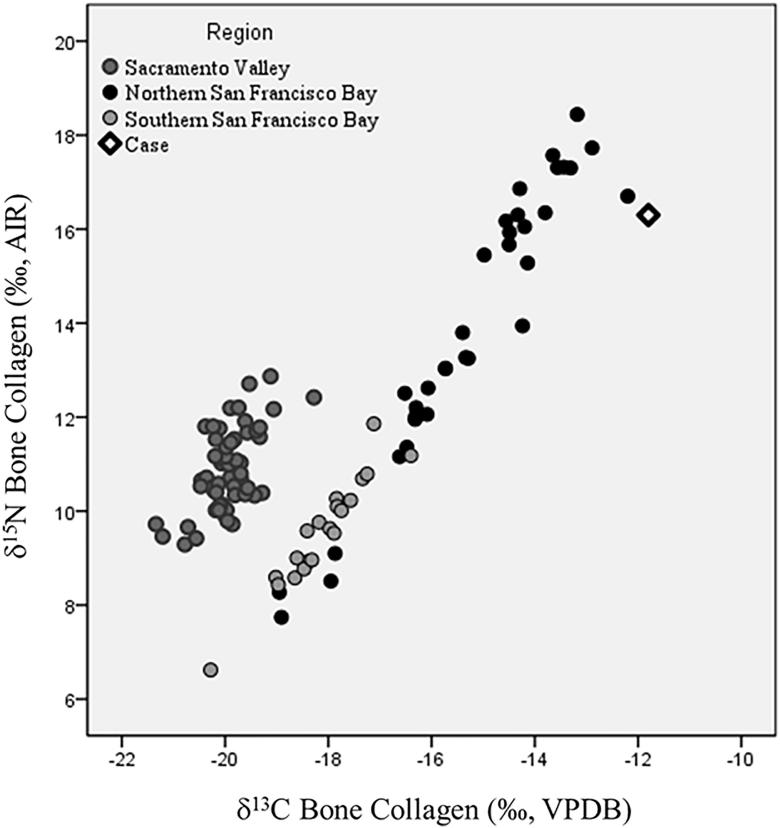
Bivariate plot of bone collagen stable carbon and nitrogen isotope values of prehistoric hunter-gatherers from Central California. Unidentified bone sample (labeled as “Case”) plots closest to San Francisco Bay Area Native Americans from the northern San Francisco Bay Area. AIR: Atmospheric Nitrogen; VPDB: Vienna-Pee Dee Belemnite.

Given that the macroscopic and microscopic analyses were inconclusive (but suggestive of human), the construction firm approved the use of isotope analysis to determine whether or not the bone collagen had carbon and nitrogen isotopic compositions similar to prehistoric Native Americans from the San Francisco Bay Area. If the bone was a prehistoric human from the northern Bay Area, we expected high *δ*^13^C and *δ*^15^N values similar to those found in other nearby sites, reflecting a diet composed of high-trophic level marine protein. Figure 2 plots the *δ*^13^C and *δ*^15^N values of bone collagen from the sample compared to prehistoric hunter-gatherers from the northern and southern San Francisco Bay Area and Central Valley of California [98–100]. Isotopic compositions of the unknown bone sample are closest to prehistoric humans from the northern San Francisco Bay Area, including a burial (*n* = 1) from a nearby San Francisco site (CA-SFR-175) and also sample of 18 burials from a shellmound (CA-ALA-307) located just on the opposite shore of the Bay from San Francisco. The elevated *δ*^13^C and *δ*^15^N values indicate heavy consumption of high-trophic level marine resources, such as marine and estuarine fish. The only other mammals with elevated *δ*^13^C and *δ*^15^N values are marine mammals, including the sea otter, California sea lion, and Northern fur seal [98–100]. However, macroscopic characteristics can exclude sea mammals as the source of the bone fragment. While the isotope data in itself cannot be used to definitively identify an unknown bone as human, it can provide strong circumstantial evidence. In this case, the coroner’s office and construction firm accepted the results as support for the bone belonging to a prehistoric Native American.

#### Case Study 2: identifying travel history of an unidentified human remains case

In 2010, a human skull was located in the desert in southern California. The biological profile indicated the remains were those of a young Hispanic female. Despite obtaining a DNA profile and conducting an extensive missing persons search, law enforcement was unable to identify the decedent. In 2017, the remains were submitted to the HIL for preparation and isotope analysis. Investigators were interested in knowing whether the decedent may have been from Latin America versus a U.S. born Hispanic individual. Second, they requested information on whether isotope data from hair could shed light on the decedent’s travel history in the months prior to death. Carbon isotope data from bone and tooth enamel bioapatite suggested a mixed C_3_/C_4_ diet, more consistent with U.S. Americans than Latin Americans. Given this information, we suggested that the decedent was likely from the continental U.S. To track her most recent movements, 10–12 hair strands were sampled; they were oriented at the basal (root) to distal ends. Hair samples were cleaned to remove debris and contaminants by rinsing twice for 5 min each in a solvent mixture (2:1 chloroform: methanol). After cleaning, the hair strands were taped at the root end into a clean tinfoil pouch and submitted for measurement of oxygen isotope ratios. The bundle of hair strands was cut into segments using a razor blade. Oxygen isotope ratios were measured for 14 serial sections (approximate total length of 15–16 cm) of the decedent’s hair, representing approximately 14 months before death. Segment lengths were measured using calipers. A uniform growth rate of 0.39 mm/d was applied to measured segment lengths to calculate relative dates. Each month of the year was defined as 30.5 d. This enabled an estimation of the time period represented by each hair segment, with time zero being the basal (root) end of the hair, representing the time closest to death. Segments that were isotopically very similar – indicating little variation in drinking water inputs during the associated time periods – were averaged.

**Figure 3.**
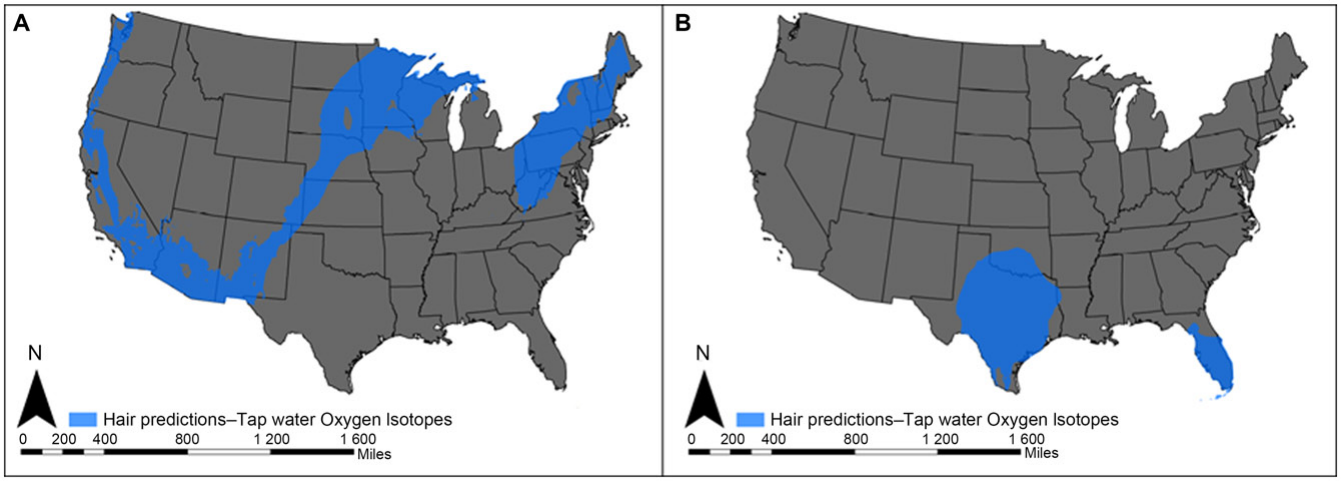
**(A**) Region-of-origin prediction map for Case Study 2 using the oxygen isotopic composition of hair. (Water base layer data used for region-of-origin prediction from [20].) The highlighted areas indicate locations where the decedent may have obtained her drinking water (based on measured oxygen isotope ratios of hair) between 13 and 3 months prior to death (Region 1). These possible regions include areas within the Western U.S. (Washington State, Oregon, California, southern Nevada, Colorado), the Southwest (Arizona, New Mexico), the Midwest (Kansas, Nebraska, the Dakotas, Minnesota, Michigan), the Mid-Atlantic States, West Virginia, and most of the Northeast. (**B**) Region-of-origin prediction map for Case Study 2 using the oxygen isotopic composition of hair. (Water base layer data used for region-of-origin prediction from [20].) The highlighted areas indicate locations where the decedent may have obtained her drinking water (based on measured oxygen isotope ratios of hair) between approximately 3 and 1 months prior to death (Region 2). These possible regions include areas within the South (Texas, Oklahoma) and South Atlantic (Florida). (Maps created by E.L. Kipnis, IsoForensics, Inc.).

Figure 3A is an isoscape prediction map of Region 1, showing the possible locations where the decedent may have obtained her drinking water between 13 and 3 months prior to death. Figure 3B is an isoscape prediction map showing the possible locations where the decedent may have obtained her drinking water during the last 3 months prior to death, referred to as Region 2. The large difference in the isoscape predictions between Region 1 and Region 2 suggests a shift in residence patterns during the last 3 months of life. While the location where the remains were found cannot be ruled out as a possible region-of-origin between 13 and 3 months prior to death (Region 1; Figure 3A), the decedent appears to have spent time in a much warmer region during the last 3 months of life (Region 2; Figure 3B). The isotope results in this case was useful in suggesting the decedent was most likely from the U.S. (not Latin America), and that she traveled to a warmer region prior to death. These data can provide new investigative leads that may ultimately result in personal identification of the decedent.

#### Case Study 3: identifying undocumented border crossers from the Texas-Mexico border

A recent increase in migrant border crossings through South Texas has resulted in a rise in the number of migrant deaths along the Texas-Mexico border, which by 2011 exceeded the number of migrant deaths along the Arizona-Mexico border [101]. Increased border security has also created a “funnel effect” in which migrants are forced to cross through more inhospitable and dangerous terrain [102–104]. Migrant deaths are most often caused by heat-related illness and dehydration [102,104], with the majority of fatalities occurring around the Falfurrias checkpoint in Brooks County (Texas), located approximately 80 miles north of the border. In 2013, forensic anthropologists from Baylor University and University of Indianapolis began the process of exhuming human remains of deceased unidentified border crossers (UBCs) buried at a cemetery in Brooks County. The remains are housed at Texas State University’s FACTS, where they are currently being analysed, identified, and repatriated to families. Known as “Operation Identification”, this work involves collaboration between multiple institutions and NGOs in a concerted effort to identify these deceased migrants and repatriate them to their next-of-kin in Latin America.

**Figure 4.**
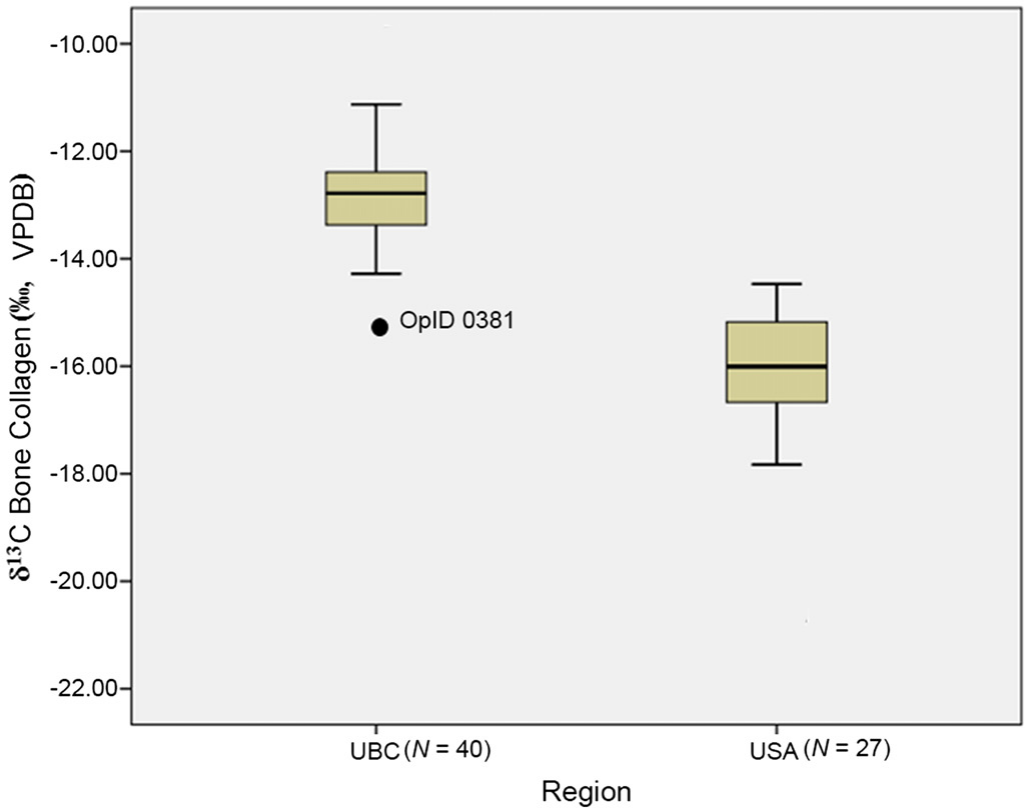
Box-and-whisker plot comparison of bone collagen stable carbon isotope data for a sample of UBC remains from South Texas and a sample of U.S. Americans (partial data set derived from [105], Figure 15.1, P. 179). Box represents the interquartile range (IQR) and whiskers are 1.5 × IQR. Unidentified bone sample (labeled as “OpID 0381”) is more similar to U.S. Americans than other UBC remains. VPDB: Vienna-Pee Dee Belemnite. UBC: unidentified border crosser; USA: U.S. American.

Between 2016 and 2018, teeth and bone samples of UBCs were provided to the HIL from Texas State University for preparation and isotope analysis. For one of these individuals (OpID 0381), a male recovered from Falfurrias, Texas, we sampled a premolar and metatarsal to obtain information on provenance and diet, respectively. Bone collagen data indicate a diet composed of both C_3_ and C_4_ resources. However, as shown in Figure 4, the *δ*^13^C value (−15‰) of the collagen was surprisingly lower than that found in other UBC remains, and is more similar to reported data on U.S. Americans (Figure 15.1 in Ref. [105], P.179).

**Figure 5.**
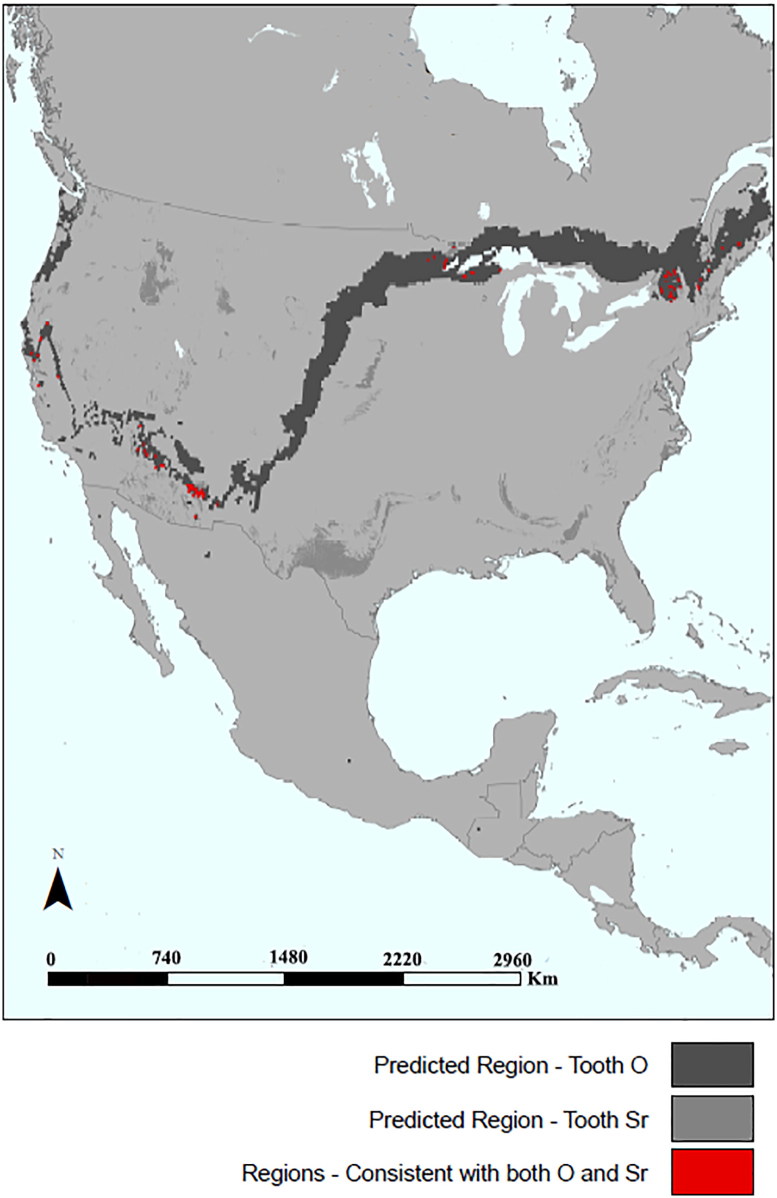
Region-of-origin prediction map for Case Study 3, remains of an unidentified border crosser (OpID 0381), using the oxygen and strontium isotopic compositions of tooth enamel. (Water base layer data used for region-of-origin prediction from [20] and [60].) The darkest gray highlighted areas indicate locations where the individual may have obtained their drinking water (based on measured oxygen isotope ratios of tooth enamel). The lighter gray highlighted areas indicate locations where the individual may have obtained their food (based on measured strontium isotope ratios of tooth enamel). The red highlighted areas indicate locations where both oxygen and strontium isotopic compositions overlap, representing the most likely regions-of-origin. These possible regions include several areas within the Western U.S., the Southwest, the Midwest, and the Northeast. (Map created by B.J. Tipple, IsoForensics, Inc.).

Figure 5 illustrates an isoscape prediction map of the possible locations where the decedent may have obtained his drinking water and food during childhood. The darkest gray highlighted areas indicate locations where he may have obtained drinking water (based on oxygen isotopes). Similarly, the lighter gray highlighted areas indicate locations where he may have obtained food (based on strontium isotopes). The red highlighted areas indicate locations where both oxygen and strontium isotopic predictions overlap, representing the most likely origin. Oxygen and strontium isotope data based on tooth enamel provide region-of-origin predictions for several areas, including the Western U.S. (California), the Southwest (Arizona and New Mexico), the Midwest (Minnesota and Wisconsin), and several states in the Northeast. However, predictions based on the oxygen isotope value (δ^18^O = –6.9‰) unexpectedly excluded both Mexico and Central America. Although there currently is very little modern strontium baseline data available for Latin America, the ^87^Sr/^86^Sr ratio of 0.70686 is not inconsistent with bioavailable strontium within the Motagua Valley in southern Guatemala and also western Honduras based on data from a number of bioarchaeological studies [106–108]. Based on these isotope data, these areas cannot be excluded as a possible place of origin for the decedent. However, it is a strong possibility that the decedent was U.S. born unlike the vast majority of UBCs. There are at least three possibilities: (1) the decedent is not a UBC but was treated as such in death; (2) the decedent may have spent his childhood years in the U.S. but may have been deported and died on his return journey; or (3) the decedent may be from an area within Latin America that is poorly characterized isotopically. Additional research is needed to more fully address cases such as this that diverge from the expected pattern of other UBC remains analysed to date.

## Future research directions

It is important that future applications of isotope analysis consider possible roadblocks to progress in forensic anthropology casework. First and foremost is the paucity of nationally or internationally agreed and validated standard operating procedures (SOPs) for the preparation and analysis of human remains (teeth, bone, hair, and nails). In addition, the community needs relevant reference data of sufficient quantity and quality to support interpretation/evaluation of tissue isotopic compositions. Unfortunately, funding constraints can limit the basic or applied research necessary to develop SOPs and reference datasets that are fit for purpose.

There are additional challenges to introducing isotope data as evidence in a court of law, including the lack of recognized matrix-matched reference materials or reference materials in appropriate matrices to report data traceable to the isotope ratio *δ* scales – for example, collagen and bioapatite from bone. Presenting interpreted data in ways that are most useful to law enforcement is another important consideration when introducing isotope testing results to the legal system. Practitioners may need predictive models describing isotopic variation in materials of forensic interest that do not yet exist or exist only on small scales (e.g. H isotopes of collagen, Pb isoscapes, etc.).

Finally, the case studies presented herein highlight the fact that a multi-isotopic profile is almost always beneficial for forensic anthropology casework as opposed to analysis of only one or two isotope systems. In other words: “The more isotopes, the merrier!” [109]. Analysis of other isotope ratios (e.g., B, Li, and Mo) may prove useful for forensic purposes in the future, although they do not yet have widespread application in medicolegal cases. Addition of these other isotope systems to forensic anthropology casework will likely require development of new instrumentation – as well as new reference materials.

## Conclusion

While isotope analysis has been used for several decades to determine whether samples of chemically similar substances – such as drugs, explosives, paints, plastics, or tapes – may share a common source, applications of the technique to unidentified human remains for forensic profiling purposes are continuing to emerge. Here, we attempted to provide a brief but comprehensive overview of isotope analysis and its utility to medicolegal cases. Although it is impossible to cover all facets of isotope analysis for human remains testing in a single article, the background, case studies, and references included in this work provide a foundation for forensic anthropologists interested in using this scientific tool to provide investigative leads in their own casework.
